# Activity of apremilast in a patient with severe pemphigus vulgaris: case report

**DOI:** 10.3389/fimmu.2024.1404185

**Published:** 2024-06-25

**Authors:** Cheyenne Delvaux, Gérôme Bohelay, Ishaï-Yaacov Sitbon, Isaac Soued, Marina Alexandre, Joël Cucherousset, Laurent Gilardin, Antoine Diep, Frédéric Caux, Christelle Le Roux-Villet

**Affiliations:** ^1^ Department of Dermatology, Saint-Pierre and Brugmann University Hospitals, Université Libre de Bruxelles, Brussels, Belgium; ^2^ Department of Dermatology and Referral Center for Autoimmune Bullous Diseases MALIBUL, Avicenne Hospital, AP-HP, Sorbonne Paris Nord University, Bobigny, France; ^3^ Department of ENT, Avicenne Hospital AP-HP, Sorbonne Paris Nord University, Bobigny, France; ^4^ Department of Pathology, Avicenne Hospital AP-HP, Sorbonne Paris Nord University, Bobigny, France; ^5^ Department of Internal Medicine, Jean-Verdier Hospital, Bondy, France; ^6^ Department of Immunology, Saint-Louis Hospital, Paris, France

**Keywords:** apremilast, pemphigus vulgaris, auto-immune bullous disease, keratinocytes, phosphodiesterase 4 inhibitor

## Abstract

**Introduction:**

Although the treatment for pemphigus vulgaris (PV) has been revolutionized by the use of rituximab combined with corticosteroids, new effective therapies with a better safety profile are needed.

**Observation:**

A 67-year-old woman was diagnosed with severe mucosal PV, which was initially misdiagnosed as atypical Behçet’s disease. Following an unsuccessful colchicine treatment, significant improvement was observed upon the introduction of apremilast: reduced pain, fewer lesions, and a stabilized weight. The discontinuation of apremilast led to a rapid relapse. Retrospective analysis through anti-Dsg3 ELISA indicated a gradual decrease in antibody levels during the apremilast treatment.

**Discussion:**

Apremilast, a phosphodiesterase 4 inhibitor approved for psoriasis and Behçet’s disease’s related oral ulcers treatment, demonstrated its efficacy in this PV case. This is the second case report highlighting the effectiveness of apremilast for PV treatment. Apremilast’s ability to upregulate cyclic adenosine monophosphate (cAMP) levels appears to contribute to the stabilization of keratinocyte adhesion.

**Conclusion:**

Apremilast may be a promising therapeutic option for the treatment of pemphigus, with an innovative mechanism of action, no induced immunosuppression, and good tolerance. It could be a good alternative to steroids, in the treatment regimen of steroids combined with rituximab.

Pemphigus vulgaris (PV) is a severe autoimmune blistering disease affecting mucous membranes and skin. Lesions are caused by a loss of adhesion between keratinocytes in the epithelium due to anti-desmoglein auto-antibodies (1). Formerly treated by systemic corticosteroids and sometimes by immunosuppressants such as azathioprine or mycophenolate mofetil, its management has been revolutionized by the use of rituximab. First used in combination with steroids as a second line for refractory pemphigus, studies showed at the time that treating pemphigus early in the disease course led to better remissions rate (2, 3). Thus, rituximab associated with systemic corticosteroids is now recommended in first line for moderate to severe PV (4). One of the current challenges of PV treatment is to reduce its associated side effects; notably, low doses of rituximab have been proposed in the literature to improve the safety profile (5). But new effective therapies with a better safety profile are needed.

We present the case of a 67-year-old Tunisian woman with conjunctival erythema and very painful recurrent oral and esophageal erosions responsible for severe dysphagia and a 10-month progressive 22 kg weight loss. She had a history of high blood pressure, type 2 diabetes and dyslipidemia. Oral lesions were misdiagnosed as aphthous ulcers, and the patient was initially treated for an atypical Behçet’s disease, first with colchicine at 1 mg per day during 2 months without improvement. Colchicine was then replaced by apremilast, administered orally by increasing the doses gradually over 1 week up to a dose of 30 mg twice daily. After 3 weeks of apremilast use, a significant improvement was observed, with reduced pain rated from 8 to 4 by the patient on a numeric rating scale (NRS, 0–10), and fewer lesions. This allowed the resumption of a solid diet along with weight stabilization for the first time in ten months. A few days later, the patient was referred to our center for autoimmune bullous diseases (AIBD) for evaluation because of ongoing mild dysphagia and oral lesions. The first clinical exam by our stomatologist revealed the presence of six mildly painful oral erosions (pain score of 3), and no aphthous ulcers were observed. Biopsies of the oral mucosa and ELISA/immunological tests were performed, followed ten days later by a multidisciplinary evaluation which revealed oral lesions had healed but laryngeal erosions and ocular erythema remained. No other abnormalities, notably no skin lesions, were detected. Apremilast was well tolerated but it was discontinued as lesions evoked an AIBD rather than Behçet’s disease. This discontinuation led to a rapid relapse after a few days with new painful oral lesions, pharyngeal and laryngeal erosions (**Figure 1**), choanal crusts and non-fibrosing conjunctivitis despite the prednisolone mouthwashes (prednisolone 20 mg soluble tablets, twice a day). Awaiting laboratory investigation results and considering its previous efficacy and its favorable safety profile, apremilast was resumed off-label at 30 mg twice daily, resulting again in significant improvement of the pain with a quick cessation of new oral lesions within a few days (**Figure 2**). Histology revealed intraepithelial suprabasal cleavage and acantholysis; direct immunofluorescence showed IgG deposition in a fishnet-like pattern and anti-desmoglein (Dsg) 3 antibodies ELISA was positive (81 UI/ml, N < 20). Anti-Dsg1 ELISA was negative. A diagnosis of severe PV with ocular, oral, nasal, laryngeal, pharyngeal and esophageal involvement was established. Once the diagnosis of PV was confirmed and despite the clear improvement under apremilast, it was decided to treat the patient with a classic regimen of rituximab and corticosteroids, according to the last French and European guidelines (4). She received a treatment with prednisone 1 mg/kg/day in combination with rituximab (1g at two-week intervals) and apremilast was finally discontinued, although she was reluctant to stop it. Complete clinical remission was obtained two months later; no adverse event was reported. Anti-Dsg3 ELISA was retrospectively performed on former sera showing a gradual decrease of its levels, beginning while she received apremilast alone (**Figure 2**).

**Figure 1:**
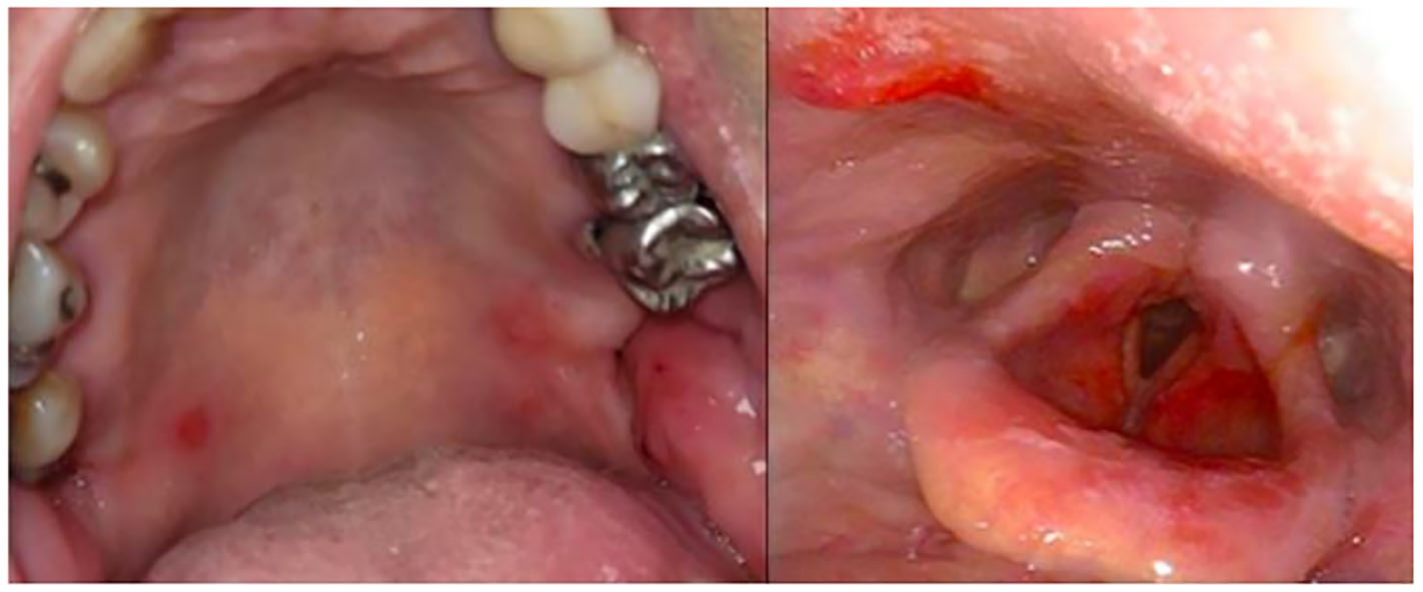
Oral and laryngeal erosions after apremilast discontinuation.

**Figure 2:**
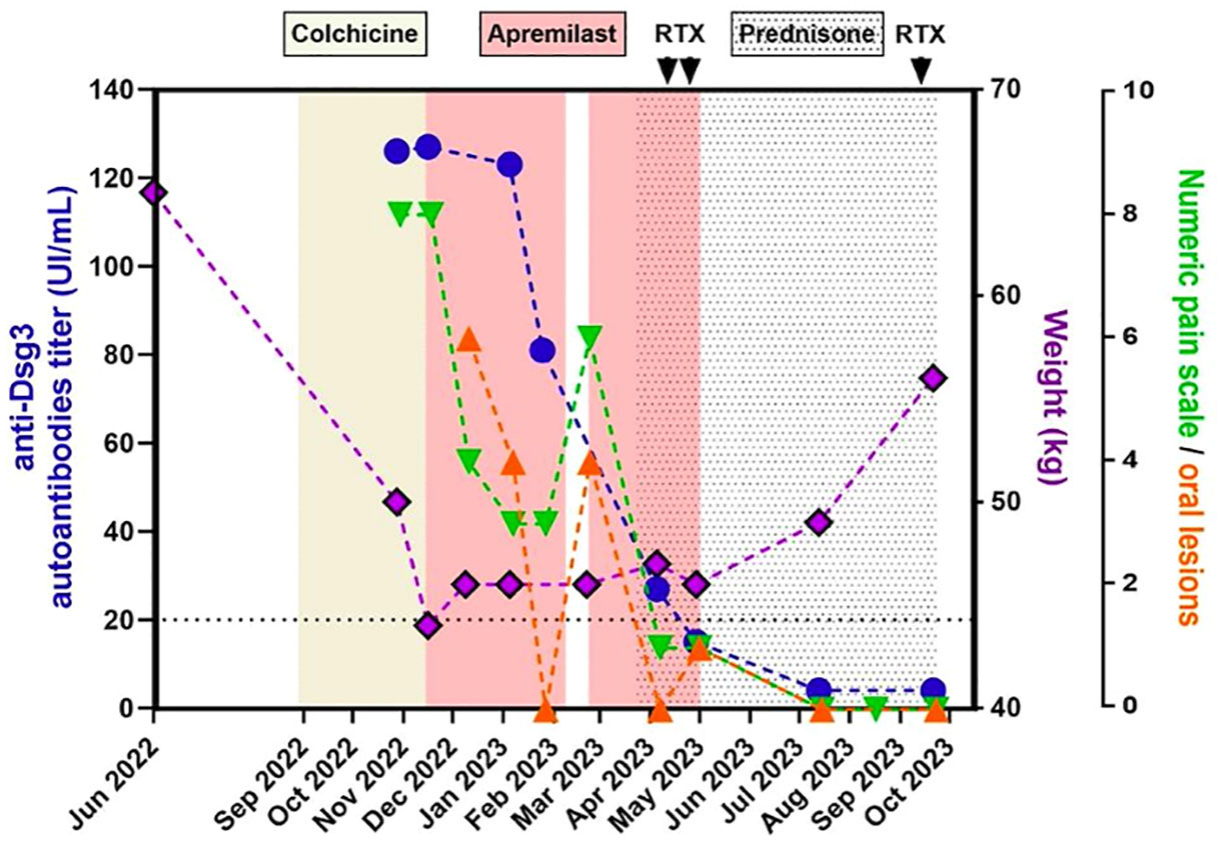
Evolution over time of anti-Dsg3 autoantibodies titer, weight, numeric pain rating scale and number of oral lesions.

Apremilast, a phosphodiesterase 4 (PDE4) inhibitor, was approved several years ago for plaque psoriasis and psoriatic arthritis and more recently for oral aphthous ulcers in patients with Behçet’s disease (6, 7). In our case, apremilast resulted in the healing of lesions as well as a decrease of the pain NRS from 8 to 3, demonstrating an improvement of clinical parameters and a progressive one of immunological parameters. Moreover, the quick clinical relapse after apremilast cessation and the rapid improvement after its reintroduction also suggest its efficacy in PV treatment. This is the second reported case of apremilast’s effectiveness in PV after the article from 2020 by Meier et al, which showed the quick healing of lesions and a decrease of anti-Dsg antibody levels under apremilast treatment (8). Interestingly, clinical trials of apremilast in Behçet’s disease showed a particularly rapid effect on pain levels and the number of oral ulcers, as we could observe in our patient (7). Apremilast is known to increase intracellular cyclic adenosine monophosphate (AMPc) levels and thus to down-regulate some pro-inflammatory cytokines such as Il-17 and INF-γ via the activation of the protein kinase A. Meier et al. also observed a continuous increase in T cell regulatory levels during PDE4 inhibition, while autoantibody levels decreased (8). Moreover, a recent study showed that apremilast, by enhancing AMPc levels in keratinocytes, could also prevent blistering in human epidermis and could stabilize keratinocyte adhesion in PV (9). In addition, two clinical cases reported apremilast’s efficacy in subepidermal autoimmune bullous diseases: a case of anti-p200 pemphigoid associated with psoriasis, and a case of conventional treatment-resistant IgA linear dermatosis (10, 11). In the latter case, authors suggest that a part of the activity of apremilast may be mediated by its inhibitory effect on IL-8 production and thus on chemotaxis of neutrophils, cells which play a key role in the mechanism of IgA linear dermatosis.

Unlike steroids or rituximab, no severe infections were related to apremilast which is usually well tolerated: the most frequent side effects, which are gastrointestinal disturbances and headaches, usually disappear after a few weeks of treatment.

In conclusion, apremilast seems to be a very interesting molecule for the treatment of pemphigus, with an innovative mechanism of action, no induced immunosuppression, and good tolerance. It could be a good alternative to steroids in a combined treatment with rituximab. A proof-of-concept study should be performed to confirm the promising early effects noted in these two cases.

## Data availability statement

The original contributions presented in the study are included in the article/supplementary material. Further inquiries can be directed to the corresponding author.

## Ethics statement

Written informed consent was obtained from the individual(s) for the publication of any potentially identifiable images or data included in this article.

## Author contributions

CD: Writing – original draft. GB: Writing – review & editing, Supervision, Methodology. ISi: Writing – review & editing. ISo: Writing – review & editing. MA: Writing – review & editing. JC: Writing – review & editing. LG: Writing – review & editing. AD: Writing – review & editing. FC: Writing – review & editing. CLR-V: Writing – review & editing.
